# Successful development of novel monoclonal antibodies against HTLV-1 bZIP factor and their applications in studying the pathogenesis of HAM/TSP

**DOI:** 10.1186/1742-4690-8-S1-A244

**Published:** 2011-06-06

**Authors:** Mineki Saito, Reiko Tanaka, Akira Kodama, Toshio Matsuzaki, Masahito Suehara, Yuetsu Tanaka

**Affiliations:** 1 Department of Immunology, University of the Ryukyus, Okinawa, 903-0215, Japan; 2 Department of Neurology, Ohkatsu Hospital, Kagoshima, 890-0067, Japan; 3 Department of Neurology, National Okinawa Hospital, Okinawa, 901-2214, Japan

## Background

A recent report has indicated that transgenic expression of HTLV-1 bZIP factor (HBZ) in CD4+ T cells induced T-cell lymphomas and systemic inflammation in mice. However, there is limited information on in vivo expression of HBZ in HTLV-1 infected individuals especially HAM/TSP.

## Methods

We have generated rat and human monoclonal antibodies against HBZ by hybridoma methodology and a phage display system, and developed the ELISA system for the detection of HBZ protein and anti-HBZ antibodies. Using these reagents, we analyzed the expression of HBZ protein in plasma and peripheral blood mononuclear cells (PBMCs) of HTLV-1 infected individuals as well as HTLV-1 infected cell lines. Then the results were compared with the real time PCR data.

## Results

Although we successfully detected HBZ protein in an enforced overexpressed cells and HTLV-1 infected cell lines by flow cytometry, ELISA, immunofluorescence and immunoblotting, we could not detect HBZ protein or anti-HBZ antibodies in HAM/TSP patients and asymptomatic carrires (ACs) by our system. In contrast, HBZ mRNA was significantly elevated in HAM/TSP patients than ACs. Especially, rapidly progressive HAM/TSP patients showed extremely high expression of HBZ mRNA but not tax mRNA in their PBMCs.

## Conclusions

These findings suggest that although expression of HBZ protein was suppressed in vivo in HAM/TSP patients and ACs, high expression of HBZ mRNA is closely associated with the pathogenesis of HAM/TSP.

